# Hospital and Institutional News

**Published:** 1918-11-02

**Authors:** 


### HOSPITAL AND INSTITUTIONAL NEWS.

### RECENT DEATH OF A HOSPITAL CHAIRMAN.

Though it was for two years only that the late Sir Scott Foster held the chairmanship of the Royal Portsmouth Hospital, he had been connected with the institution since 1901. After becoming a vice-president in that year, the year of his mayoralty, he joined the board of management in 1902. Retiring three years later, he rejoined in 1907, and was appointed vice-chairman in 1914, a position which he held for two years, till his promotion to the chairmanship. His institution, by the way, is an instance of the fact that wards are kept closed nowadays not on the false premiss of want of funds, but on the real and unescapable ground of want of staff. Mr. T. H. F. Lapthorn, the chairman of the meeting at which Sir Scott Foster's death was officially recorded, has succeeded in persuading his colleagues to appoint a committee to inspect the proposed home for paraplegics and discharged convalescents at Purbrook Park, for which the Ministry of Pensions has desired the hospital to be responsible. The new chairman is Mr. Woolmer White, who has acted as treasurer in the past.

### THE LATE DAME WESTON AND THE NAVY.

Now that the nineteenth century is beginning to gain the perspective of history we see that its institutional growth was marked by a steady line of "philanthropists," beginning with Mrs. Fry, of humorous memory. The details can still be studied, and they are often amusing, in the works of the late Mr. Kirkman Gray. Of the Mrs. Fry lineage, Dame Agnes E. Weston was one, and, like others of the sisterhood, she lived to a great age. Her death on October 24 removes the better known of an energetic pair of women, the other being Miss Weston's coadjutor, Miss Wintz. When twenty-eight years old, in 1868, Miss Weston began visiting hospitals. Band of Hope and Sunday school activity followed, and a letter to a man on H.M.S. *Crocodile* started her well-known work for the Navy. She was almost what Nordau would call a graphomaniac, and the extent of her correspondence appals those who take a conservative view of the value of time spent on writing letters. So she had soon to adopt the method of the circular, and soon these printed communications flooded the Navy. The response seemed to show that they were appreciated, notwithstanding the colour given to their contents by Miss Weston's interest in the Temperance movement. She founded the Naval Temperance Society, which is said to have a branch now in every ship in the senior Service. The establishment of Sailors' Rests followed, and the relative cheerfulness and convenience of these places overcame any objection which might have been felt in respect to the drinks sold therein. Sleeping accommodation later on was added for the families of the men. There was a regular room for "evangelistic" and other meetings; classes, clubs clustered round. The experiment at Devonport led to its initiation on the same lines but on a bigger scale at Portsmouth. The work won the Royal approval. Miss Weston was duly canonised by her admirers as the "Sailors' Friend," and every reader of The Hospital has probably received one of her multitudinous appeals or circulars at one time or another. In June last she received the G.B.E., and her work survives among the many kindred efforts of voluntary enthusiasts in the field of philanthropy.

### THE LATEST MOVE AT CARDIFF.

There is luckily no end to the devices which alert hospital managers can employ in the creation of sectional subscriptions. Works, areas, villages, classes, town and country, have all been placed at one time or another in friendly competition. So have the seven ages of man for seven different types of patients; in fact, the voluntary system itself is nothing but the organisation of sectional subscriptions from the well for the benefit of the sick. This principle is well understood in Wales, where its application is vigorous and varied. Colonel Bruce Vaughan has now started a body of "almoners"—women, that is to say, who have undertaken to raise a number of recruits and a certain sum of money on behalf of the King Edward VII. Hospital's new maternity department at Glossop Terrace, Cardiff. This is the League of Mercy plan over again. When, however, the first enthusiasm has waned it is difficult to prevent the organisers of these bodies from debasing the original idea—that, namely, of personal service—into a vulgar catchword, and from holding the view that the object of the particular body is to raise money, and that once a few pious phrases have been vented it does not much matter how the cash is "roped in."

### THE END OF ALL CRUSADES.

This is the danger which time brings to all such bodies, from the Salvation Army to the League of Mercy itself. Beginning with enthusiasm they are apt to end in cant, and their organisers to degenerate from men of public spirit to 'cute advertisers and piously disguised commercial sharks. The process is seen in the change which is apt to come over the procedure of these bodies. They begin with addresses, which inspire adherents. But as the first band of enthusiasts disperses, the addresses become fewer; there is, perhaps, none capable of giving them. Funds tend to fall. An organiser (that fetish of commercialism) is hired at a certain salary. He probably regards the motto of the body which employs him as a respectable decoration for his note-paper, but a correct but meaningless platitude in itself. He is after the cash. Consequently he resorts to bazaars, and the more or less respectable shifts of commerce. The funds may be maintained; but the body which first evoked them will have changed from an inspired band of apostles to a bureaucracy of business men. At this stage the voluntary spirit dies of inanition and the work performed by the body itself is ripe to be taken over by the rates. It is because the beginnings at Cardiff have been so good that it is vital to utter the warning of experience before the danger, which may come, has supervened.

### RESIGNATION OF SIR THOMAS R. FRASER.

The Royal Infirmary, Edinburgh, has lost by resignation the services of Sir Thomas R. Fraser, M.D., F.R.S., Professor of Materia Medica and Therapeutics, and of Clinical Medicine at Edinburgh University. Senior physician at the Royal Infirmary, Sir Thomas began his connection with it in 1860, when he became assistant physician. Ten years later he had completed his first year of professorial duty, and in all has been for forty years in charge of the medical wards. He has now been made consulting physician to the Royal Infirmary.

### THE DAWSON IDEAL.

We note elsewhere the heart-searchings now active in Bradford concerning the future of the scheme for building a great new infirmary there. The opponents of the voluntary system, as their way is, have been active in suggestion, and, on the well-worn cry that the voluntary system has "broken down," propose, in the person of Mr. R. S. Dawson, a municipal alternative. As chairman of the Bradford Board of Guardians, Mr. Dawson's views on municipal questions deserve attention. But when he trespasses on the province of the voluntary system he will pardon its adherents for maintaining a critical, though attentive, reserve. His plan, briefly, is "after the war" for the Guardians to hand over St. Luke's Hospital to the Health Committee of the Bradford Corporation. In addition, he suggests that a hospital sub-committee of the Health Committee should be established to control all the Health Services of the city, and "to subsidise, if necessary, the existing voluntary hospitals." The sub-committee, according to his idea, should be composed of municipal officials and an (unspecified) number of representatives of the medical profession and of the voluntary hospitals. The weak point of the latter part of the proposal is the express assumption on which it is based—namely, the voluntary hospitals at present "stand altogether apart from aid and control by public authorities." On the contrary, the facts are that the public authorities have thrust so much work upon the voluntary hospitals that, to protect themselves and the classes of patients for whom they cater, the hospitals have been compelled to ask for a grant in aid of the extra work which they have agreed to do, and in consequence to receive some representatives of the public authority which subsidises them. Therefore Mr. Dawson's proposal is one which puts the cart before the horse. It is for the public authorities to submit representatives to the hospitals, not for the hospitals to be represented upon the public authority. With the grave exception thus established, the details of Mr. Dawson's ideal can be considered with some hope of avoiding confusion. Meanwhile, why should the scheme for a new infirmary in Bradford be held up?

### A DOCTOR'S WARNING TO "THE AFFLUENT WORKING MAN."

Since our exhaustive summary in The Hospital of the principles involved in the dispute between the working men of Swansea and the medical staff with reference to the men's demands for what may be called disproportional representation, it might seem that little could be added. Since then, however, Dr. Jabez Thomas has written a remarkable letter to the local press, in which he says that, if the working men are to rush through their proposal regardless of all other considerations but their own, the hospital has its remedy. It can, he says in effect, decline the working men's subscriptions. He therefore gives to them the following message: "Take your money and go away, and leave us in peace; means will be found to carry on the work of this great hospital for the sick and needy. The affluent working man of these days can look after himself." Here at last is the note of leadership, which, whether this frank policy be acted on or not, rouses a response in every breast capable of rising to an emergency. It is indeed time that hospital managers in industrial districts should scrutinise carefully the claims of every would-be patient, no matter to what class he may boast himself to belong. It is time, too, for the more responsible chairmen and treasurers to think twice before accepting any subscription from any source which seems to be given in the spirit, not of charity, but of commercialism.

### THE RED CROSS IN RUSSIA.

Lord Robert Cecil has explained, in answer to inquiries, that both the British and the American Red Cross Societies, together with the Y.M.C.A. of both countries, have "arranged" to send contingents to Archangel, Murmansk, and Vladivostock. To Northern Russia, it seems, medical supplies have already been dispatched, and we hope that that which has been "arranged" will also arrive in adequate amount, and not too long after it is needed.

### NEW SUPERINTENDENT OF ABERDEEN ASYLUM.

Dr. R. Dods Brown, F.R.C.P., physician superintendent of James Murray's Royal Asylum, Perth, has been appointed superintendent of the Royal Asylum, Aberdeen, at a salary of £1,000 a year. After graduating in medicine in 1902 at Edinburgh, Dr. Brown was appointed resident physician in the Royal Infirmary, Edinburgh, and in 1904 transferred in a similar capacity to the Edinburgh City Hospital for Infectious Disease. A year later he took the D.P.H., and was one of the first to gain the diploma of psychiatry at Edinburgh. Appointed by the late Sir James Clouston assistant physician to the Royal Edinburgh Asylum, in 1908 he took charge of the 600 patients in West House, and eventually became deputy physician superintendent. It is now five years since he became physician superintendent to James Murray's Royal Asylum, Perth, where he has contributed many technical papers to learned societies and professional journals.

### THE BOBBY'S BAN.

The appeal for "Our Day" this year was complicated by the refusal of the Public Carriage Branch of the police, according to Mr. C. F. Higham, to permit "bills of appeal" to appear on the omnibuses. It was stated that the ground of the refusal was that the work of the Red Cross was not of national importance, but we can hardly credit any official, outside a Government Department, finding such an exquisite excuse. The facts are worth an explanation, which the new Chief Commissioner, General Sir Nevil Macready, to save the credit of the Force, would be wise in detail to provide.

### THE NEW INFIRMARY, BRADFORD.

Although land has been purchased for the proposed new infirmary for Bradford, and plans even have been drawn, alternative suggestions seem to be having a certain vogue in the district. One is for the conversion of St. Luke's Hospital, which has many of the defects complained of in the present Royal Infirmary. Since some of the money needed has already been promised or raised, it is time that those responsible for the original scheme should give a lead to public opinion.

### THE SHAKESPEARE HOSPITAL.

The recent opening at Glasgow of the Shakespeare Hospital is interesting apart from its dedication, for the object is to provide "indoor treatment" for discharged disabled men of both Services. In short, we are glad to see it is now recognised that the term "discharged" is not a term of precision, and that by discharging a man the authorities have by no means finished with their obligations to the ex-fighter. The chief of the resident staff at the Shakespeare Hospital is Dr. Taylor, and the opening ceremony was graced by that veteran Sir George Beatson, who has a fine record of work to his credit.

### A HOSTEL FOR PATIENTS' FRIENDS.

Among many instances of thoughtfulness for the sick and wounded the following is worthy of mention. A lady living close to one of the general military hospitals in the Midlands has thrown open her house as a hostel for friends of patients on the danger list who have been summoned, perhaps from a long distance, in order, possibly, to say a last farewell to son or husband. These visitors frequently arrive at a time which makes their return that night impossible, they are then taken to the hostel, looked after, fed and lodged for the night; no charge is made, all the expenses, we understand, are borne by the lady referred to, who also personally looks after those who come.

### REDUCED PENSIONS FOR MENTAL STAFFS?

At a recent meeting of the County Councils' Association, held at the Guildhall, Westminster, a letter was received from the Central Association for the Care of the Mentally Defective criticising the effect which the provisions of the Asylums and Certified Institutions (Officers' Pensions) Act, 1918, will have on the staffing of certified institutions under local authorities. It appears that the staffs of these certified institutions will be regarded as of secondary importance, and will have smaller pensions. The consequence will probably be that local authorities will have difficulty not only in getting new officials, but in retaining their existing staffs. The Home Office is being attacked with a view to the amendment of the Act.

### WHERE ARE THE CORONERS?

In reference to the matter raised by a correspondent in The Hospital of September 28, Dr. Waldo's statement at a recent inquest is of much interest. In respect to the case in question, the City Coroner said that he disagreed with the Registrar who was reported to have attributed decrease in the number of such cases to decrease in the amount of liquor consumed by the parents. Such a cause, in his (the Coroner's) experience, was quite a minor one, and the slight increase in the number of such deaths on Saturday to Sunday mornings and on public holidays was mostly due to the overworked condition of the poor mother, followed by sounder and heavier sleep, and the consequent more frequent death of the infant from pressure and suffocation. He (the Coroner) had never held an inquest in such class of case without ordering a *post-mortem* examination, which not infrequently resulted in proof of death from natural, and not violent, causes. Broncho-pneumonia, congenital defects of heart, status lymphaticus (as in case before jury), etc., was often disclosed by the autopsy. Needless and unfair stigma was sometimes cast on the parent which might be saved by means of an autopsy. Dr. Waldo's point appears to be that the stigma of "drink" complained of by our correspondent would be removed by the practice of holding an autopsy. So far so good. But is it not a fact that autopsies are often opposed by the class of patients affected?

### V.D. WARD AT HUDDERSFIELD.

The board of Huddersfield Royal Infirmary has decided to reserve certain beds for the treatment of male and female cases of venereal disease. The staff in charge is to include a woman doctor. A series of meetings has begun to acquaint the district with the nature of the work and the need for it, and to secure support for an important addition of usefulness to the Royal Infirmary.

### MANCHESTER'S NEW HOSPITAL.

The committee of the East Lancashire Homes for Disabled Sailors and Soldiers have temporarily transferred to the War Office "Grange Thorpe," in Rusholme, for an orthopædic centre. The War Office has agreed to build extra wards. The curative workshops also will be provided, and an appeal for £25,000 is being made. This will be the first orthopædic hospital in Manchester, and it is practically ready for its work.

### THE ITALIAN WAY.

The *Domenica del Corriere* states that the populace of Ponte Albiate—an industrial region of the Brianza—has been holding festival in honour of a modest benefactress of the poor, Signorina Giuseppina Vigano, who is, while still a young woman, at the head of a large and wealthy industry. She has always given largely. Without mentioning other conspicuous benefactions, she has recently given 620,000 lire (£26,000) to the Hospital of Victor Emmanuel Third of Carate Brianza for convalescent soldiers. All the people of the neighbourhood participated in the festival in her honour, which was attended by the civil and military authorities.

### THE PROVISION OF RED CROSS DOWRIES.

The Red Cross flag now waves triumphantly over every kind of organisation. It is a universal provider. But ever new needs are being pressed upon its attention. According to the *American Red Cross Bulletin*, a request was lately received by the American society to provide, so to speak, a *dot* for an intending bridegroom. The appeal was sent by a Belgian soldier from a town behind the Front. It ran: "I am on the point of getting married, and should like to know whether you could help me slightly in a financial way, as all my relatives are in invaded Belgium, and the only money I have is my army pay. My *fiancée* is as poor as I am. She is a refugee at St. Brieux." The editor of the *Bulletin* discreetly leaves us in exquisite suspense concerning the fate of this very human and pathetic appeal. We hope that, if a friendly investigation of the circumstances warrants, someone may come to the rescue, for whether Red Cross funds, even of American origin, are legally able to be used to provide dowries is necessarily a nice point. On the other hand, the writer and his *fiancée* have added another proverb to the language: and when we want to excite sympathy we should no longer say "as poor as a church mouse," but rather "as poor as a Belgian refugee." Alas! that Belgium should ever have become a proverb in this respect.

### CLINICAL THERMOMETERS.

An Order has been made by the Ministry of Munitions which establishes a minimum degree of error for clinical thermometers. The Order requires that all clinical thermometers, before sale, must be sent to the National Physical Laboratory for testing and approval, and up to November 11 next no such thermometer will be approved if it shows at any point in its registration of temperature an error of more than 4° F., that after November 11 no such thermometer will be approved if it shows an error exceeding 2° F. over the range up to 106° F., and that no thermometer will be approved if it is not self-registering, with a constriction which must be such as to retain the index column and also allow of the mercury being reset. It is all to the good that clinical thermometers should be as reliable as it is possible to make them, but the Order above noted is another example of Departmental callousness. In Government Departments we are so accustomed to inordinate delays that we have reached the point when we expect delay almost as a right. But here we have the opposite extreme. We are informed that, had this Order come into operation at the appointed time, the effect would have been that nobody, neither doctor nor nurse, would have been able to buy a clinical thermometer for several weeks. This was pointed out to the proper authority by the Pharmaceutical Society, and as a result the Order will not be enforced before November 21. It is a pity that Government Departments do not make an invariable rule of consulting experts before making orders affecting technical and business affairs.

### DUTCH MEDICAL TRADITION.

It will probably interest our readers in connection with the action recently tried (see The Hospital, June 29, 1918, page 282), that it is in Holland, quite different from what we are accustomed to, a good old traditional use among the medical profession to accept no fees from fellow-practitioners on account of medical attendance, without mentioning the presents they make each other. It is true that in the main "two of a trade seldom agree," but the practice mentioned contributes largely to the fact that in general the Dutch medical men act as good colleagues to each other, and that such objectionable cases as the action of Dr. Nicholls against Mr. de Santi do not occur in Holland.

### WHERE THE PORTER SETS THE PACE.

It certainly does not seem unreasonable that nurses should be anticipating some improvement in their pay and in other respects when one comes across advertisements of this kind (the cottage hospital referred to is in South Wales, but we have omitted the name of the place): "Wanted, porter for cottage hospital; usual duties plus driving motor ambulance; salary 50s. weekly with rations and uniform, or 70s. without rations." If the porter is paid £2 10s. a week (or £3 10s. without rations) at this hospital, how much ought the matron to receive? In all probability her salary is no more than the porter's—possibly it may be less.

### THIS WEEK'S DRUG MARKET.

The swift developments in the political and military situation are slow in affecting the drug market, but business for the most part is restricted to immediate requirements, and although there is no general downward tendency in prices the tone of the market is not altogether firm. Of course, the influenza epidemic has stimulated the demand in many parts of the world for certain drugs such as quinine, eucalyptus oil, aspirin, phenazone and phenacetin, and the volume of business in these has been considerable. At the end of last week the price of eucalyptus oil began to sag, but this week there has been another small boom, notwithstanding that stocks here are good. In view of the present comparatively small supplies of quinine available, the value is well maintained in spite of the impending Government control. Menthol is quieter, but the price has not given way to any noteworthy extent.

